# Structural and microvascular changes of the peripapillary retinal nerve fiber layer in Von Hippel–Lindau disease: an OCT and OCT angiography study

**DOI:** 10.1038/s41598-020-79652-w

**Published:** 2021-01-08

**Authors:** Elisabetta Pilotto, Elisabetta Beatrice Nacci, Gilda De Mojà, Alfonso Massimiliano Ferrara, Raffaele Parrozzani, Davide Londei, Stefania Zovato, Edoardo Midena

**Affiliations:** 1 Department of Ophthalmology, University of Padova, Padova University Hospital ERN-EYE Center, Padova, Italy; 2 IRCCS, Fondazione G. B. Bietti, Via Livenza 3, 00198 Rome, Italy; 3 Familial Tumor Unit, Veneto Institute of Oncology IOV-IRCCS, Padua, Padova Italy

**Keywords:** Cancer screening, Predictive markers

## Abstract

Von Hippel–Lindau (VHL) disease is an autosomal dominant genetic disease caused by VHL gene mutation. Retinal hemangioblastomas (RH) are vascularized tumors and represent the main ocular manifestation of the disease. Histopathologically, RH are composed of capillary vessels and stromal cells, the neoplastic population of the lesion. The origin of these stromal cells remains controversial, even if they are hypothesized to be glial cells. The aim of the present study was to investigate neuronal and microvascular changes of the peripapillary retinal nerve fiber layer, in which glial cells, neurons and capillaries (the radial peripapillary capillary plexus) interact. VHL patients with or without peripheral RH were enrolled and compared to healthy controls. Mean peripapillary retinal nerve fiber layer (pRNFL) thickness was measured by means of optical coherence tomography (OCT). The following vascular parameters of the radial peripapillary capillary plexus were quantified using OCT angiography: Vessel Area Density,Vessel Length Fraction, Vessel Diameter Index and Fractal Dimension. One hundred and nine eyes of 61 patients, and 56 eyes of 28 controls were consecutively studied. Mean pRNFL was significantly thinner in VHL eyes without RH versus eyes with RH and controls. Mean pRNFL thickness did not differ between VHL eyes with RH and controls. All OCTA vascular parameters were reduced in VHL eyes with or without RH versus controls, with significative difference for Vessel Diameter Index. The same OCTA parameters did not significantly differ between VHL eyes with or without RH. In VHL eyes without RH, pRNFL thinning may be the consequence of impaired perfusion of the radial peripapillary capillary plexus, while the increase of pRNFL thickness in VHL eyes with RH may depend on possible activation and proliferation of the other RNFL resident cells, the glial cells.

## Introduction

Heritable cancer syndrome Von Hippel–Lindau (VHL) is a genetic disease caused by VHL gene mutation^1^. VHL gene encodes a tumor suppressor protein (pVHL) which forms a VHL protein complex responsible for the degradation of hypoxia-inducible factors (HIFs). In absence of pVHL, HIFs increase with subsequent over-production of numerous growth factors, up-regulates angiogenesis and increases cellular proliferation. Pro-angiogenic and pro-mitotic stimuli lead to the development of various tumors including hemangioblastomas, typical vascularized hamartomatous lesions that develop in the central nervous system and in the retina^2,3^. Retinal hemangioblastoma (RH) originate in the neurosensory retina or optic disc. They are histopathologically similar to central nervous system hemangioblastomas^1^ and composed of capillary vessels and stromal cells, the latter representing the neoplastic population^2,4^. The origin of these stromal cells remains controversial, even if they are hypothesized to be glial cells^2,5–10^. The advent of optical coherence tomography (OCT) and OCT angiography (OCTA) in clinical practice allows to explore single retinal layers and their vascularization, without any dye injection^11^. The aim of this research was to analyze, using OCT and OCTA, the structural and perfusion characteristics of the peripapillary retinal nerve fiber layer, in which glial cells, neurons and capillaries (the radial peripapillary capillary plexus) interact, in VHL eyes with or without peripheral RH.

## Results

### Population characteristics

From December 2018 to January 2020 sixty-one caucasian VHL patients and 28 heathy controls underwent enrolment. The groups were homogeneous for mean age (39.0 ± 13.8 years vs 40.0 ± 11.8, p = 0.7373) and sex distribution (female/male: 32/28 vs 17/11, p = 0.5591). In 13 patients only one eye was evaluable, while fellow eye was excluded because of: retinal detachment (1 case), presence of RH at the posterior pole or optic disc (5 cases), band keratopathy (2 cases), epiretinal macular membrane (3 cases), macular retinal pigment epithelium changes (1 case), myelinated nerve fibers (1 case). Therefore, 109 VHL eyes of 61 patients (38 eyes with RH [34.8%] and 71 without RH [65.1%]), and 56 eyes of 28 healthy subjects were included.

### Peripapillary retinal nerve fiber layer thickness analysis

Mean Peripapillary Retinal Nerve Fiber Layer (pRNFL) thickness was significantly thinner in VHL eyes without RH compared to controls (100.7 ± 13.4 μm vs 104.2 ± 7.4 μm, p = 0.0172). No difference was found between VHL eyes with RH and controls (103.5 ± 9.9 μm vs 104.2 ± 7.4 μm, p = 0.7421) as pRNFL thickness is concerned. Mean pRNFL thickness was significantly thicker in VHL eyes with RH versus those without RH (103.5 ± 9.9 μm vs 100.7 ± 13.4 μm, p = 0.0201). In all temporal sectors, sectorial pRNFL was thicker in eyes with RH versus those without RH (73.3 ± 9 μm vs 66.2 ± 11.1 μm, p = 0.0002 for Temporal; 147.4 ± 18.4 μm vs 134.9 ± 18.3 μm, p = 0.0005 for Temporal Superior; 152.2 ± 20.9 μm vs 145.9 ± 22.4 μm, p = 0.0083 for Temporal Inferior; 55.1 ± 7.1 μm vs 51.1 ± 7.1 μm, p = 0.001 for Papillo-Macular Bundle). Thinning of sectorial pRNFL in eyes without RH versus controls mostly involved the temporal sectors (p = 0.0454 for Temporal and p = 0.0127 for Temporal Superior). A detailed sectorial analysis of pRNFL thickness of all studied eyes is reported in Table 1.

**Table 1:** Peripapillary retinal nerve fiber layer (pRNFL) thickness in VHL eyes with or without RH and healthy controls.

pRNFL thickness (μm: mean ± SD)	VHL w/out RHs (71 eyes)	Healthy controls (56 eyes)	VHL with RH (38 eyes)	VHL w/out RH vs controls *p-value*	VHL with RH vs VHL w/out RHs *p-value*	VHL with RH vs controls *p-value*
Mean pRNFL	100.7 ± 13.4	104.2 ± 7.4	103.5 ± 9.9	**0.0172**	**0.0201**	0.7421
T-pRNFL	66.2 ± 11.1	71.7 ± 14.2	73.3 ± 9.0	**0.0454**	**0.0002**	0.5334
TS-pRNFL	134.9 ± 18.3	144.6 ± 16.3	147.4 ± 18.4	**0.0127**	**0.0005**	0.6526
NS-pRNFL	116.1 ± 31.7	112.4 ± 19.7	117.2 ± 27.9	0.6076	0.6338	0.4462
N-pRNFL	78.5 ± 18.8	81.9 ± 13.4	76.3 ± 15.6	0.3735	0.7145	0.1471
NI-pRNFL	119.3 ± 26.1	121.4 ± 17.8	111.2 ± 14.5	0.6727	0.0880	**0.0226**
TI-pRNFL	145.9 ± 22.4	150.9 ± 19.0	152.2 ± 20.9	0.2036	**0.0083**	0.7188
PMB	51.1 ± 8.8	52.9 ± 7.4	55.1 ± 7.1	0.3429	**0.0010**	**0.1714**

N-pRNFL, nasal sector-peripapillary Retinal Nerve Fiber Layer; NI-pRNFL, nasal-inferior sector-peripapillary Retinal Nerve Fiber Layer; NS-pRNFL, nasal-superior sector-peripapillary Retinal Nerve Fiber Layer; PMB, papillo-macular bundle; RH, retinal hemangioblastomas; T-pRNFL, temporal sector-peripapillary Retinal Nerve Fiber Layer; TI-pRNFL, temporal-inferior sector-peripapillary Retinal Nerve Fiber Layer; TS-pRNFL, temporal-superior sector-peripapillary Retinal Nerve Fiber Layer; VHL, Von Hippel Lindau disease; w/out, without.

Significant p value in bold.

### OCT-angiography vascular indexes analysis

**Figure 1:**
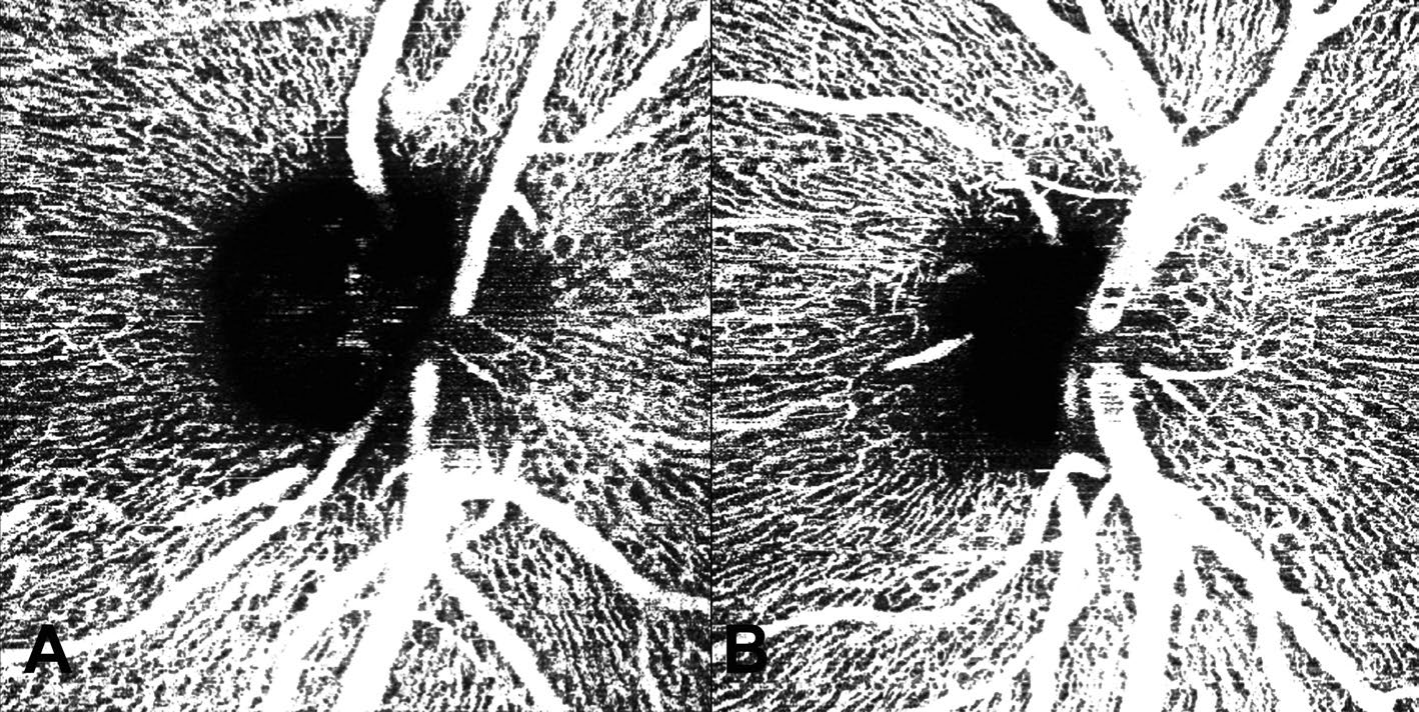
Peripapillary OCT-Angiography capillary plexus in VHL eye (**A**) and Healthy control (**B**). En face OCT-Angiography of the Radial Peripapillary Capillary Plexus showed reduced vascular perfusion in a VHL eye (**A**) compared to a healthy control (**B**). Quantification of plexus perfusion, expressed through evaluation of Vessel Area Density (VAD), Vessel Length Fraction (VLF), Vessel Diameter Index (VDI), Fractal Dimension on binary image (FDbin) and Fractal Dimension on skeletonized image (FDsk) indexes, detected a significant reduction in VHL eyes of VDI parameter.

All OCT-Angiography (OCTA) vascular parameters, including Vessel Area Density (VAD), Vessel Length Fraction (VLF), Vessel Diameter Index (VDI) and Fractal Dimension (FD), were reduced in VHL eyes with and without RH versus controls, reaching statistical significance for VDI (p = 0.0365 and p = 0.0293, respectively). Figure 1 “Peripapillary OCT- Angiography capillary plexus in VHL eye (A) and Healthy control (B)”.

OCTA vascular parameters did not significantly differ between VHL eyes with or without RH. (Table 2).

**Table 2:** OCT angiography parameters of the Radial Peripapillary Capillary Plexus (RPCP) in VHL eyes with or without RH and healthy controls.

RPCP mean (SD)	VHL w/out RH (71eyes)	Healthy controls (56 eyes)	VHL with RH (38 eyes)	VHL w/out RH vs controls *p-value*	VHL with RH vs VHL w/out RHs *p-value*	VHL with RH vs controls *p-value*
VAD	0.4881 (0.13)	0.52390 (0.09)	0.5059 (0.12)	0.1248	0.4630	0.4609
VLF	0.0853 (0.02)	0.0889 (0.01)	0.0885 (0.01)	0.2830	0.5038	0.9281
VDI	5.6312 (0.64)	5.8827 (0.53)	5.6747 (0.60)	**0.0293**	0.7529	**0.0365**
FDbin	1.8182 (0.09)	1.8466 (0.04)	1.8346 (0.06)	0.0929	0.6281	0.2860
FDsk	1.5357 (0.07)	1.5568 (0.04)	1.5539 (0.05)	0.1628	0.4333	0.7518

FDbin, Fractal Dimension of binary image; FDsk, Fractal Dimension of skeletonized images; RH, retinal hemangioblastomas; VAD, Vascular Area Density; VDI, Vessel Diameter Index; VHL:Von Hippel-Lindau Disease; VLF, Vessel Length Fraction.

Significant p value in bold.

## Discussion

In the present study, the peripapillary retinal nerve fiber layer was thinner in VHL eyes without RH, both compared to controls and to eyes with RH. At OCTA analysis of the radial peripapillary capillary plexus, the Vessel Diameter Index, which refers to the diameter of the vessels^12^, was significantly reduced in VHL eyes compared to healthy controls, independently of the presence or absence of RH, showing a plexus characterized by analomalous thin capillaries. The radial peripapillary capillary plexus is a distinct retinal vascular network, located in the pRNFL, characterized by a specific radial distribution of capillaries, tightly coupling and suppling the nerve fibers^11,13,14^. Retinal blood flow is highly correlated to neural activity, in a strict neurovascular metabolic coupling^15^. Therefore, the reduced perfusion of the this plexus may be correlated to the reduction in thickness of the RNFL, as occurs in other retinal diseases like diabetes and glaucoma, whose neurodegenerative nature is well demonstrated^13,16–18^. In a retina-specific knockout VHL animal model, excessive vessel reduction and regression have already been found^19^. Therefore, an impaired radial peripapillary capillary plexus, characterized by reduction in peripapillary perfusion, may hypothetically cause reduction of retinal nerve fibers.

On the other hand, in VHL eyes with RH, showing the same radial peripapillary capillary plexus perfusion impairment, pRNFL was thicker than in eyes without RH. Therefore, radial peripapillary capillary plexus changes may be excluded in the pathophysiology of this relative thickening. The retinal nerve fiber layer is composed not only by nerve fibers and vessels, but also by glial cells, mostly Müller cells and astrocytes^20,21^(from 18 to 42%). Moreover, the thickness of pRNFL has been shown to be linearly correlated to the number of retinal astrocytes^20^. The role of astrocytes in VHL disease remains controversial, but histopathologic studies have already revealed trasformed and increased astrocytes in the stromal populations of hemangioblastomas, including the retinal ones^5,9,10,22–24^. Under normal conditions, astrocytes maintain the homeostasis of the nervous tissue –controlling, protecting and supporting neuronal function- and regulate local blood flow^25^. Under pathological conditions, atrocytes react through a process called reactive astrogliosis, with hyperplasia/proliferation of the cells, increased number of their processes and larger cell body size^25,26^. In an experimental model, the deletion of VHL gene in retinal atrocytes induced not only increased astrocyte cell number in the RNFL, but also upregulation of retinal VEGF production, which is a key factor in the pathophisiology and growth of hemangioblastomas^27,28^. Therefore, the relative increase in pRNFL thickness we detected in eyes with RH and not in eyes without RH, represents in our view human in vivo clinical evidence of local activation of retinal astrocytes with consequent upregulation of retinal VEGF production, previoulsy demonstrated in animal models and in hystopathological studies.

In VHL patients, OCT and OCTA have been mainly used to study posterior pole RH or macular capillary changes related to RH^29–32^. To the best of our knowledge, this is the first OCT and OCTA study investigating the structural and perfusion characteristics of the peripapillary nerve fiber layer, in which glial cells, neurons and capillaries of the radial peripapillary capillary plexus interact.

Genotype–phenotype correlations were not investigated in the present research. This could represent a limit of the study, since we cannot exclude that the genotype of VHL germline mutation may differently influence the analyzed parameters, as higher prevalence of progression-related complications and preretinal fibrosis have already been correlated to it^33,34^. A further analysis of genotype versus OCT and OCTA parameters is ongoing.

In conclusion, in VHL eyes, pRNFL is thinner than in controls hypothetically as a consequence of reduced perfusion of the radial peripapillary capillary plexus, as shown in other retinal disorders. When peripheral RH are present, pRNFL thickness relatively increases compared to eyes without RH. This fact may be due to the proliferation of retinal astrocytes, as previously histologically reported. This observation may offer a holistic interpretation of the involvement of different retinal cells in VHL progressive ocular disease. A clinical application of our findings may be that, in the follow-up of VHL patients, the increase of pRNFL thickness, compared to baseline, may guide retinal examination for peripheral RH screening. Larger population and longer follow up are mandatory to confirm our observations.

## Methods

This was a cross-sectional study and informed consent was obtained from each subject; data collection was compliant with the tenets of the Declaration of Helsinki. The approval from the Ethics Committee for Clinical Practice of the Azienda Ospedaliera di Padova for the study was obtained (Prot.34971/AOP/2018). VHL patients, referred by the Familial Tumor Unit of the Veneto Institute of Oncology (IOV-IRCCS), underwent scheduled eye examination including: best-corrected visual acuity (BCVA) measurement using standard Early Treatment Diabetic Retinopathy Study (ETDRS) charts, anterior segment evaluation at slit lamp, intraocular pression measurement, fundus examination by means of indirect ophthalmoscopy and 90-diopter-lens biomicroscopy. Patients who presented congenital or acquired anterior segment diseases; epiretinal macular membrane; posterior pole and optic nerve – treated or untreated RH; glaucoma; refractive errors ≥ 6 diopters; history of ocular inflammation; concomitant presence of other retinal vasculopathies were not included in the research. The posterior pole and optic nerve RH were excluded because they may interfere with the correct visualization and quantification of RNFL and vascular parameters. Moreover, patients with pituitary stalk hemangioblastomas, which may compromise optic nerve pathway, were also excluded^35^. An age-matched healthy group was recruited for comparison.

### OCT and OCT angiography

After pupil dilatation (obtained with 1% tropicamide eye-drops solution), enrolled eyes performed OCT and OCTA using Spectralis HRA + OCTA (Heidelberg Engineering, Heidelberg, Germany) in a semi-dark room, late in the morning and with the in-built eye-tracker always active to obtain high quality scans and correct foveal centration of the scans. The OCT scan protocol included a peripapillary 3.5 mm ring N-RNFL (N-site/axonal menu) scan, centered onto the optic nerve head, to automatically provide the peripapillary retinal nerve fiber layer (pRNFL) thickness value. pRNFL thickness was expressed both as mean pRNFL (pRNFL) and as sectorial pRNFL (Temporal, T-pRNFL; Temporal Superior, TS-pRNFL; Nasal Superior, NS-pRNFL; Nasal, N-pRNFL; Nasal Inferior, NI-pRNFL; Temporal Inferior, TI-pRNFL; Papillo-Macular Bundle, PMB).

An OCTA scan pattern of 10° × 10° (3.0 × 3.0 mm; 512 B-scans separated by 6 micron) centered onto the optic nerve head was acquired. The inbuilt software automatically generated the enface OCTA image of the radial peripapillary capillary plexus, which is the radial capillary monolayer network encircling the optic disc^11,13^. As previously described^29^ in order to guarantee high quality OCTA images, only images with a signal strength more than 30 in “Q score” (on a scale of 0 to 40 for Spectralis, Heidelbeerg), were analysed^36,37^. Correct foveal centration of the scans was obtained with the inbuilt eye tracking system. A skilled technician checked each image after acquisition to detect any motion artifacts or segmentation errors, and eventually repeated examination. Open-source available ImageJ software (National Institutes of Health, Bethesda, MD, USA) allowed quantitative analysis of the OCTA en face images, obtaining quantitative parameters of the radial peripapillary capillary plexus: Vessel Area Density (VAD), Vessel Length Fraction (VLF), Vessel Diameter Index (VDI) and Fractal Dimension (FD), as previously described^12,38^. OCTA en face images were automatically converted into a binary image. VAD, which provides the estimate of real vessel density in the examined area, taking into consideration both vessel length and vessel diameter, was obtained dividing the number of black pixels in the binary image by the total number of image pixels. The binarized image was then “skeletonized” deleting the outer boundary of the binarized image so that each vessel segment had a single pixel width. By dividing the number of vessel pixels in the skeletonized image by the total number of image pixels VLF was obtained, quantifing the vessel length regardless of the vessel diameters. VDI parameter, sensitive to vascular dilation in the OCT-A images as it presents the vessel size information regardless of the vessel length, was obtained by processing both the binary and the skeletonized images to calculate the average vessel caliber. FD, representing complexity of images, was calculated both over the skeletonized images (FDsk) using a box counting technique and over binary images (FDbin)^12,38^.

### Statistical analysis

All variables were summarized according to the conventional methods of descriptive statistics: mean and standard deviation for quantitative variables; absolute and relative (percentage) frequencies for qualitative variables. Normal distribution of parameters was checked by Shapiro–Wilk’s test.

Gender distribution and age of enrolled patients and controls were compared using chi-square test and t-Student test for independent samples, respectively. Mean pRNFL and sectorial pRNFL thickness values were analyzed with one-way ANOVA model. When applicable, statistical models were adjusted for replication of measures in both eyes of patients and controls.

A mixed effects ANOVA model was applied to compare OCTA parameters between groups, with repeated measures (both eyes), and adjusted for age. Statistical significance was set at p < 0.05 level. All the analyses were performed by SAS® 9.4 statistical software (SAS Institute, Cary, NC, USA).

## Data Availability

The authors have full access to all the data and take responsibility for the integrity of the data and the accuracy of the data analysis as well as the decision to submit to publication.
